# An Amino Acids Mixture Improves the Hepatotoxicity Induced by Acetaminophen in Mice

**DOI:** 10.1155/2013/615754

**Published:** 2013-06-26

**Authors:** Francesco Di Pierro, Giuseppe Rossoni

**Affiliations:** ^1^Scientific Department, Velleja Research, Viale Lunigiana 23, 20125 Milan, Italy; ^2^Department of Medical Biotechnology and Translational Medicine, University of Milan, Via Vanvitelli 32, 20129 Milan, Italy

## Abstract

Acetaminophen (APAP) is a widely used analgesic and antipyretic drug, but at high dose it leads to undesirable side effects, such as hepatotoxicity and nephrotoxicity. The aim of this study was to evaluate the protective role of DDM-GSH, a mixture of L-cysteine, L-methionine, and L-serine in a weight ratio of 2 : 1 : 1, in comparison to N-acetylcysteine (NAC), against acetaminophen- (APAP-) induced hepatotoxicity in mice. Toxicity was induced in mice by the intraperitoneal (ip) administration of low dose (2 mmol/kg) or high dose (8 mmol/kg) of APAP. DDM-GSH (0.4 to 1.6 mmol/kg) was given ip to mice 1 h before the APAP administration. The same was done with NAC (0.9 to 3.6 mmol/kg), the standard antidote of APAP toxicity. Mice were sacrificed 8 h after the APAP injection to determine liver weight, serum alanine aminotransferase (ALT), and total glutathione (GSH) depletion and malondialdehyde (MDA) accumulation in liver tissues. DDM-GSH improved mouse survival rates better than NAC against a high dose of APAP. Moreover, DDM-GSH significantly reduced in a dose-dependent manner not only APAP-induced increases of ALT but also APAP-induced hepatic GSH depletion and MDA accumulation. Our results suggest that DDM-GSH may be more potent than NAC in protecting the liver from APAP-induced liver injury.

## 1. Introduction

Acetaminophen (APAP, 4-hydroxyacetanilide), a nonsteroidal analgesic and antipyretic drug, is used for the treatment of a variety of arthritic and rheumatic conditions with musculoskeletal pain and in other painful disorders such as headache, dysmenorrhea, myalgia, and neuralgia. It is also indicated in conditions accompanied by generalized discomfort or fever, such as common cold and viral infections. APAP is considered to be safe at the therapeutic levels. However, an overdose of APAP in human is fairly common, being yearly in USA the leading cause for calls to Poison Control Centers and accounting for more than 56,000 emergency room visits, 2,600 hospitalizations, and an estimated 458 deaths [1]. The APAP overdose is often associated with acute liver failure [1, 2] and renal damage [3] in humans, as well as in experimental animals.

At therapeutic doses, APAP is metabolized via glucuronidation and sulfuration reactions occurring primarily in the liver and results in water-soluble metabolites that are excreted renally. As a result of the metabolic conversion of APAP by the microsomal CYP-450 enzyme system, a highly reactive intermediate, N-acetyl-p-benzoquinoneimine (NAPQI), is produced [4]. NAPQI directly reacts with glutathione (GSH), and at overdoses of APAP, the depletion of cellular GSH occurs. This allows NAPQI to bind to cellular proteins and initiate lipid peroxidation, leading to hepatic [4] and renal [5] injury. However, despite recognition of APAP hepatotoxicity, therapeutic options available to either treat or prevent its development are still limited. In the absence of reliable and effective modern liver protective drugs and available traditional medicines employed for the disease treatment, concerted efforts are currently channeled towards exploring complementary or alternative medicines in the disease treatment and/or prevention. Botanicals have shown tremendous potential to serve as the alternative therapeutic agents so as to counter the side effects of various over-the-counter drugs [6, 7].

DDM-GSH is a mixture of L-cysteine, L-methionine, and L-serine in a weight ratio of 2 : 1 : 1 that we assumed to be useful to optimize GSH synthesis. In fact, L-cysteine is the main limiting step to the synthesis of glutathione [8], L-methionine is considered to be the main amino acid involved in L-cysteine neosynthesis in terms of SH-group donor [9, 10], and L-serine is an important amino acid involved in L-cysteine synthesis due to its ability to donate its carbon skeleton [11]. 

The current study was designed to evaluate *in vivo* the protective effect of DDM-GSH, in comparison to the current antidote N-acetylcysteine (NAC), on APAP-induced hepatotoxicity in mice. Comparisons were made of the protective effect of equimillimolar doses of DDM-GSH and NAC on APAP hepatic toxicity to evaluate the potency of DDM-GSH.

## 2. Materials and Methods

### 2.1. Materials

DDM-GSH was developed by Velleja Research (Milan, Italy), manufactured in Procemsa Farmaceutici (Nichelino, TO, Italy), and traded in Italy as nutritional supplement from the group Pharmextracta/Omeopiacenza (Pontenure, Piacenza, Italy). APAP and NAC were purchased from Sigma-Aldrich (Milan, Italy). Detection kits for total glutathione (GSH; no. 703002) and malondialdehyde (MDA; no. 10009055) assays were purchased from Cayman Chemical Company (Ann Arbor, MI, USA), whereas the kits for alanine aminotransferase (ALT; no. 17234E) and total proteins (no. 17620) determinations were purchased from Sentinel Diagnostics (Milan, Italy). All other chemicals were of analytical grade.

### 2.2. Animals

All studies were conducted in 6- to 7-week-old male CD-1 mice weighing 25 to 30 g, which were obtained from Charles River Laboratories (Calco, Lecco, Italy). The experiments were carried in accordance with the *Guide for the Care and Use of Laboratory Animals* published by the US National Institutes of Health (NIH Publication no. 85-23, revised in 1996) and approved by the local Ethical Committee. Every effort was made to minimize animal suffering. Mice were maintained under a controlled ambient temperature (22 ± 1°C), humidity (50 ± 5%), and 12 h light/dark cycles (light on 7:00 AM to 7:00 AM). Mice were acclimated for 7 days before initiation of any procedures. Animals had free access to water and standard rodent chow (no. 4RF25; Mucedola S.R.L., Settimo Milanese, Milan, Italy) before initiation of any treatment. However, mice were fasted before treatment with APAP as indicated in the following. 

### 2.3. APAP-Induced Mortality

In the first set of experiments, to assess the mortality rate caused by APAP, 50 mice were randomly divided into five experimental groups (*n* = 10 mice/group), including saline control group. Mice were fasted overnight (16–18 h) prior to administration of different doses of APAP (1 to 8 mmol/kg ip) dissolved in sterile phosphate buffered saline (PBS, pH 7.4) warmed to 40°C. Saline control group received sterile saline only (10 mL/kg ip). All mice were observed for up to 8 h, and lethality of APAP was taken.

### 2.4. NAC and DDM-GSH Treatments before Higher Dose of APAP

In the second set of experiments, to investigate the protective effect of NAC and DDM-GSH in the APAP-induced mortality, 40 mice were intoxicated with the higher dose of APAP (8 mmol/kg ip). Animals were randomly divided into the following four experimental groups (*n* = 10 mice/group): saline treated, APAP treated, NAC pretreated plus APAP, and DDM-GSH pretreated plus APAP. Saline (10 mL/kg), NAC (3.6 mmol/kg) and DDM-GSH (1.6 mmol/kg) were given ip 1 h before APAP. All animals were fasted overnight (16–18 h) prior to administration of a single high dose of APAP. Mice were observed for up to 8 h, and lethality of APAP was taken.

### 2.5. Serum ALT Assay, and Hepatic GSH and MDA Determinations

In the third set of experiments, to compare the activity of NAC and DDM-GSH, 80 mice (*n* = 10 mice/group) were treated with different doses of NAC (0.9 to 3.6 mmol/kg ip) and DDM-GSH (0.4 to 1.60 mmol/kg). Saline (10 mL/kg), NAC, and DDM-GSH were given ip 1 h before APAP (2 mmol/kg ip). All animals were fasted overnight (16–18 h) prior to administration of a single dose of APAP. At 8 h after APAP treatment, all the survived mice were weighted and sacrificed to collect the blood from *carotis communis*. Sera were separated from plasma and stored at −20°C until analysis. The serum levels of ALT were measured using an enzymatic colorimetric kit according to manufacturer's instruction, and the results were expressed in IU/L. Moreover, the liver was quickly excised, weighed, divided into portions, snap frozen in liquid nitrogen, and stored at −70°C for biochemical analyses of total GSH and MDA. The frozen liver slices were washed in ice-cold EDTA solution, blotted, dissected to remove connective tissues, weighed, and homogenized with 10% saline. Hepatic total GSH and MDA contents were determined by Cayman's GSH and MDA assay kits according to manufacturer's instruction, and the absorbance was measured at 405 nm and 530 nm for GSH and MDA, respectively, by Wallac 1420 VICTOR2 microplate reader (Perkin Elmer, Monza, Italy). Triplicate assays were performed in each measurement, and the average counts were obtained from each individual sample. Proteins in the tissues were assayed by colorimetric kit according to manufacturer's instruction [12]. Results were expressed in *μ*mol GSH/mg protein and nmol MDA/mg protein.

### 2.6. Statistical Analysis

All values were expressed as mean ± SE (*n* = 10 mice/group). The results were evaluated by one-way analysis of variance (ANOVA) followed by a Tukey's multiple comparison test. The half-maximal inhibitory dose (ED_50_) with 95% confidence limits and the dose ratio were also calculated. Statistically significant differences between groups were defined as *P* < 0.05. All calculations were performed with the GraphPad Prism program 5.0 (GraphPad Software Inc., San Diego, USA).

## 3. Results

### 3.1. Effect of NAC and DDM-GSH on APAP-Induced Mortality

**Figure 1:**
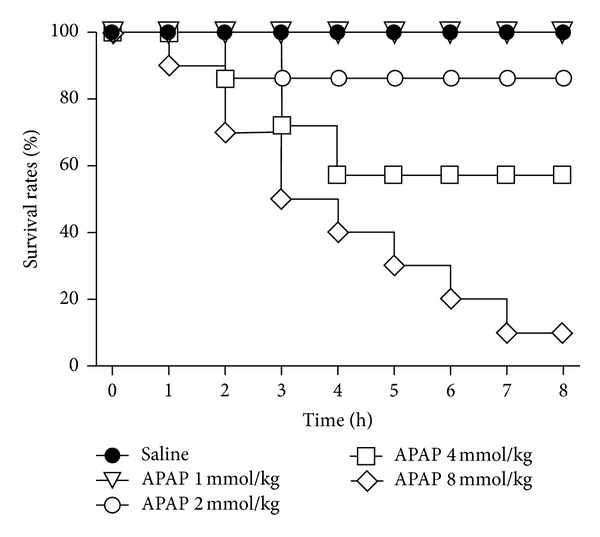
Dose relationship and time course of acetaminophen (APAP-) induced mortality in mice. Saline (10 mL/kg) and APAP were given ip. *n* = 10 mice/group.

**Figure 2:**
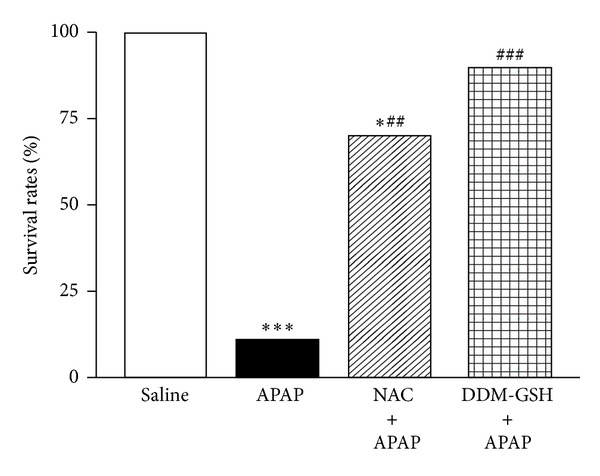
Mortality rate in mice 8 h after acetaminophen (APAP, 8 mmol/kg ip) administration, alone or in presence of N-acetylcysteine (NAC; 3.6 mmol/kg ip), and a mixture of L-cysteine, L-methionine, and L-serine in ratio 2 : 1 : 1 (DDM-GSH; 1.6 mmol/kg ip). Saline (10 mL/kg), NAC, and DDM-GSH were given ip 1 h before APAP. *n* = 10 mice/group. *P < 0.05 and ***P < 0.001 versus saline; ^##^ *P* < 0.01 and ^###^ *P* < 0.001 versus APAP.

When mice were treated with 1, 2, 4 and 8 mmol/kg ip of APAP progressive mortality rates were observed. Particularly, when the animals were intoxicated with the higher dose of APAP (8 mmol/kg ip), only a 10% of animals were survived at 8 h (Figure 1). The survival rate at 8 h was increased to 70% and 90%, respectively, when the animals were treated with NAC (3.6 mmol/kg ip) or DDM-GSH (1.6 mmol/kg ip) administered 1 h before APAP injection (Figure 2). No death was observed in the group treated with NAC or DDM-GSH alone indicating that the two compounds were safe to mice (data not shown). Considering the dosage of compounds used, these results indicated that DDM-GSH could inhibit the lethality of APAP, and this inhibition was 2-fold higher than the one observed with NAC.

### 3.2. Body Weight, Liver Weight, and Mortality Rate

When mice were treated with a low dose of APAP (2 mmol/kg ip), a 70% of animals were survived at 8 h. In these experiments, APAP increased liver weight within 8 h compared with the control (saline) group, whereas body weights were comparable between all treatment groups (Table 1). Pretreatment of the animals with NAC (0.9 to 3.6 mmol/kg ip) or DDM-GSH (0.4 to 1.6 mmol/kg ip) significantly recovered the APAP-induced mortality rate, and the increase of liver weight was reduced in a dose-dependent manner (Table 1).

### 3.3. Serum ALT Activity

**Figure 3:**
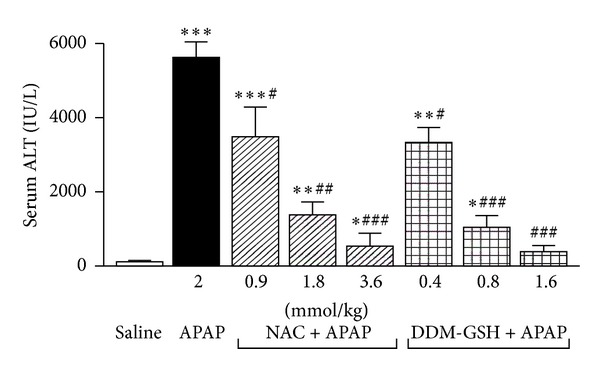
Serum alanine aminotransferase activity (ALT) in mice 8 h after acetaminophen (APAP, 2 mmol/kg ip), administration, alone or in presence of N-acetylcysteine (NAC), and a mixture of L-cysteine, L-methionine, and L-serine in ratio 2 : 1 : 1 (DDM-GSH). Saline (10 mL/kg), NAC, and DDM-GSH were given ip 1 h before APAP. *n* = 10 mice/group. *P < 0.05, **P < 0.01, and ***P < 0.001 versus saline; ^#^ *P* < 0.05, ^###^ *P* < 0.01, and ^###^ *P* < 0.001 versus APAP.

The treatment with APAP (2 mmol/kg ip) increased serum ALT activity about 70-fold at 8 h (Figure 3). Pretreatment with NAC (0.9 to 3.6 mmol/kg ip) and DDM-GSH (0.4 to 1.6 mmol/kg ip) significantly prevented, in a dose-dependent manner, the APAP-induced increase in ALT activity (Figure 3). Particularly, the protective effect of DDM-GSH (ED_50_ = 0.61 mmol/kg ip) resulted to be 2.4-fold higher (*P* < 0.01) than the one obtained with NAC (ED_50_ = 1.47 mmol/kg ip).

### 3.4. Total GSH and MDA Levels in the Liver

**Figure 4:**
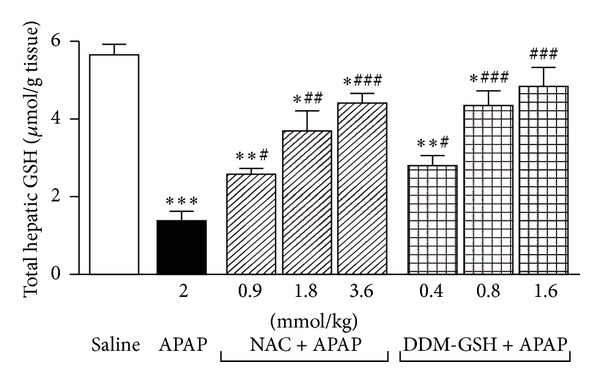
Total hepatic glutathione (GSH) in mice 8 h after acetaminophen (APAP, 2 mmol/kg ip) administration, alone or in presence of N-acetylcysteine (NAC) and a mixture of L-cysteine, L-methionine, and L-serine in ratio 2 : 1 : 1 (DDM-GSH). Saline (10 mL/kg), NAC, and DDM-GSH were given ip 1 h before APAP. *n* = 10 mice/group. *P < 0.05, **P < 0.01, and ***P < 0.001 versus saline; ^#^ *P* < 0.05, ^###^ *P* < 0.01, and ^###^ *P* < 0.001 versus APAP.

**Figure 5:**
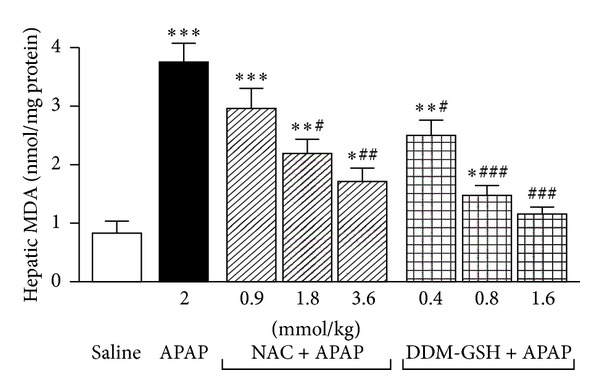
Hepatic lipid peroxidation (measured as malondialdehyde; MDA) in mice 8 h after acetaminophen (APAP, 2 mmol/kg ip) administration, alone or in presence of N-acetylcysteine (NAC), and a mixture of L-cysteine, L-methionine and L-serine in ratio 2 : 1 : 1 (DDM-GSH). Saline (10 mL/kg), NAC and DDM-GSH were given ip 1 h before APAP. *n* = 10 mice/group. *P < 0.05, **P < 0.01 and ***P < 0.001 versus saline; ^#^ *P* < 0.05, ^###^ *P* < 0.01 and ^###^ *P* < 0.001 versus APAP.

GSH plays an important role in the detoxification of APAP [13]. As reported in Figure 4, the hepatic GSH content of APAP (2 mmol/kg ip) group at 8 h decreased to 76.4% of the saline group (*P* < 0.001). Both NAC and DDM-GSH significantly recovered the APAP-induced GSH depletion in a dose-dependent manner. However, the protective effect obtained with DDM-GSH (ED_50_ = 0.68 mmol/kg ip) was 2.2-fold higher (*P* < 0.01) than that shown with NAC (ED_50_ = 1.49 mmol/kg ip) (Figure 4). The hepatic MDA content, an end product of lipid peroxidation, was increased in the APAP group (0.83 ± 0.14 nmol/mg protein) at 8 h than that in the saline group (3.75 ± 0.23 nmol/mg protein) (*P* < 0.001). The APAP-induced liver MDA increase was significantly suppressed in both NAC and DDM-GSH pretreated groups, as compared with APAP group, and this inhibition presented a dose-effect relationship (Figure 5). The protective effect of DDM-GSH (ED_50_ = 0.62 mmol/kg ip) resulted to be 2.6-fold higher (*P* < 0.01) than that obtained with NAC (ED_50_ = 1.64 mmol/kg ip).

## 4. Discussion

APAP is the most widely used over-the-counter or prescription painkiller in the world. APAP is metabolized in the liver where a toxic byproduct is produced that can be removed by conjugation with GSH. APAP overdoses, either accidental or intentional, are the leading cause of acute liver failure in the United States, accounting for 56,000 emergency room visits per year [1, 14]. GSH-depletion APAP-caused seems to be relevant not only for liver and kidney pathology, but also for asthma induction in adults [15] and in children [16] likely due to GSH depletion from bronchial and lung tissues. Its use in asthmatic subjects worsens the asthma symptoms [17]. The effect of inducing asthma seems to be provoked not only directly in APAP user but also in newborns in case their mothers have used acetaminophen during pregnancy [18]. In a recent past, legislation restricting pack sizes of acetaminophen in some countries of Europe has had substantial beneficial effects on mortality and morbidity associated with self-poisoning [19], but this strategy has not been adopted globally. The standard treatment for acetaminophen overdose is NAC, which is given to stimulate the production of GSH. With the aim of evaluating a possible improvement in terms of liver protection, we have developed a mixture, named DDM-GSH, of amino acids (L-cysteine, L-methionine, and L-serine in weight ratio 2 : 1 : 1). These has been selected on the basis of biochemical steps needed for a proper neo-synthesis of GSH, being this last a tripeptide constituted by Cys-Gly-Glu where the only real limiting step is the availability of L-cysteine and being this last synthetized from a donor of −SH (L-methionine) and a donor of the carbon skeleton (L-serine). The idea was that, not only giving L-cysteine (as with the usual treatment with NAC) but also adding two ingredients able to synthetize by their own new L-cysteine, it would be possible to reduce further the liver failure effect caused by APAP. Therefore, the objective of the present study was to compare the protective effects of NAC and DDM-GSH for APAP toxicity. The present study showed that DDM-GSH was effective at a lower dose than NAC when administering an equivalent millimolar dose. Also, these results suggest that DDM-GSH is effective in reducing APAP-induced hepatotoxicity and that DDM-GSH is likely more effective than NAC in reducing hepatic damage in the mouse model.

In 2001, from the page of BMJ, Law asked why a simple strategy like to add NAC to acetaminophen tablets or sachets for minimizing poisoning had not been enacted [20]. In all countries, NAC is registered as a drug, and from investment and regulatory point of view, this could be a limitation. NAC has anyway the advantage of being less susceptible to degradation in the gastrointestinal tract than L-cysteine and to be more readily absorbed across cell membranes. This molecular stability likely makes NAC a better L-cysteine donor for glutathione synthesis. L-cysteine, as well as L-methionine and L-serine, can however be notified all over the world as a simple dietary supplement or even used as excipient to manufacture tablets, and this is a clear advantage of L-cysteine versus NAC. Again, a disadvantage of L-cysteine is linked to its possible neurotoxic effect [21]. Clear signs of neurotoxicity were not observed at all in our investigation. Anyway, next step will be to evaluate in organs different from liver, that is, kidney, lung, and brain, the impact of DDM-GSH versus NAC in terms of tissue protection, and the evaluation of possible effects of neurotoxicity will be planned in the same experimentation. 

In conclusion, the results of our study show that likely lower doses than NAC of these amino acids are needed in mice to exert an evident protective liver effect. If the next animal investigation will show the same beneficial effects also in kidney, lung, and brain (with no neurotoxicity), we think that at least a rationale base to start checking if this effect, seen in mice, is observable in human too exists. If this was confirmed, the use of L-cysteine, L-methionine, and L-serine in formulas containing acetaminophen could reduce the risk of liver, kidney, and bronchial pathology due to acetaminophen prolonged or bad use. 

## Figures and Tables

**Table 1:** Body weight, liver weight, and survival rates in mice 8 h after administration of acetaminophen (APAP, 2 mmol/kg ip) alone or in presence of N-acetylcysteine (NAC) and a mixture of L-cysteine, L-methionine, and L-serine in a ratio 2 : 1 : 1 (DDM-GSH).

Treatment	Body weight (g)	Liver weight (g/10 g b.w.)	Survival rates (%)
Saline	23 ± 4	0.25 ± 0.01	100
APAP	24 ± 3	0.32 ± 0.01*	70*
NAC 0.9 mmol/kg + APAP	24 ± 3	0.30 ± 0.01*	90*
NAC 1.8 mmol/kg + APAP	23 ± 5	0.26 ± 0.03^#^	100^#^
NAC 3.6 mmol/kg + APAP	23 ± 4	0.26 ± 0.02^##^	100^#^
DDM-GSH 0.4 mmol/kg + APAP	25 ± 3	0.27 ± 0.02^#^	100^#^
DDM-GSH 0.8 mmol/kg + APAP	23 ± 2	0.24 ± 0.01^##^	100^#^
DDM-GSH 1.6 mmol/kg + APAP	26 ± 4	0.22 ± 0.02^###^	100^#^

Saline (10 mL/kg), NAC, and DDM-GSH were given ip 1 h before APAP. Values are reported as mean ± SE (*n* = 10 animals/group). *P < 0.05 versus saline; ^#^ *P* < 0.05, ^##^ *P* < 0.01, and ^###^ *P* < 0.001 versus APAP.
